# Functional Outcomes of a Complex Rehabilitation Program in Children with Spastic Diplegic Cerebral Palsy: A Prospective Observational Study

**DOI:** 10.3390/jcm15187092

**Published:** 2026-09-13

**Authors:** Dănuț Visarion Caimac, Anca Maria Amzolini, Simona Patru, Carmen Daniela Neagoe, Miruna Andreiana Matei, Klejda Tani, Adina Mitrea, Diana Clenciu, Amelia Valentina Genunche-Dumitrescu, Mihai Cealîcu, Paraschiva Postolache, Ana Maria Bumbea

**Affiliations:** 1 Department of Medical Rehabilitation, University of Medicine and Pharmacy of Craiova, 200349 Craiova, Romania; danut.caimac@umfcv.ro (D.V.C.); simona.patru@umfcv.ro (S.P.); anamaria.bumbea@umfcv.ro (A.M.B.); 2 Department of Medical Semiology, University of Medicine and Pharmacy of Craiova, 200349 Craiova, Romania; 3 Department of Internal Medicine, University of Medicine and Pharmacy of Craiova, 200349 Craiova, Romania; daniela.neagoe@umfcv.ro (C.D.N.); amelia.genunche@umfcv.ro (A.V.G.-D.); 4 Faculty of Medicine, University of Medicine and Pharmacy Valencia, 46010 Valencia, Spain; andreian@alumni.uv.es; 5 Department of Rehabilitation, Sport University of Tirana, 1001 Tirana, Albania; ktani@ust.edu.al; 6 Department of Diabetes, Nutrition and Metabolic Diseases, University of Medicine and Pharmacy of Craiova, 200349 Craiova, Romania; adina.mitrea@umfcv.ro (A.M.); diana.clenciu@umfcv.ro (D.C.); 7 Faculty of Medicine, University of Medicine and Pharmacy of Craiova, 200349 Craiova, Romania; mihaicealicu18@gmail.com; 8 Department of Biomedical Sciences, Grigore T. Popa University of Medicine and Pharmacy, Iasi, 700115 Iasi, Romania; par.postolache@umfiasi.ro

**Keywords:** cerebral palsy, spastic diplegia, rehabilitation, spasticity, joint mobility, gross motor function, GMFM

## Abstract

**Background:** Cerebral palsy is associated with complex motor impairments, including spasticity, reduced joint mobility, and functional limitations. Longitudinal data describing changes during sustained rehabilitation remain limited. **Objective:** We evaluated longitudinal changes in muscle tone, joint mobility, and gross motor function in children with spastic diplegic cerebral palsy participating in a structured 12-month rehabilitation program. **Methods:** This prospective, uncontrolled observational study included 94 children aged 5–10 years, selected from 116 children screened for eligibility. Assessments were performed at baseline (T0), 6 months (T1), and 12 months (T2). Outcomes included muscle tone (Modified Ashworth Scale, MAS), joint mobility (goniometry), and gross motor function (GMFM-88). Longitudinal changes were assessed using the Friedman test, followed by Bonferroni-adjusted post hoc pairwise comparisons between T0–T1, T0–T2, and T1–T2. Associations were assessed using Spearman’s rank correlation coefficient (ρ). **Results:** Significant longitudinal differences were observed across the evaluated outcomes (overall Friedman tests, *p* < 0.0001). Median MAS scores decreased from 3 at T0 to 2 at T2 across the evaluated muscle groups. Hip abduction increased, knee extension deficit decreased, and ankle dorsiflexion improved over the study period. GMFM-88 scores also increased longitudinally. Moderate-to-strong associations were observed between muscle spasticity and joint mobility parameters (ρ = −0.881 to 0.682). **Conclusions:** Favorable longitudinal changes in muscle tone, joint mobility, and gross motor function were observed during participation in the 12-month rehabilitation program. Given the uncontrolled observational design, these changes cannot be attributed exclusively to the rehabilitation intervention.

## 1. Introduction

Cerebral palsy (CP) is the most common cause of motor disability in childhood, with an estimated global prevalence of 2–3 cases per 1000 live births [1,2]. It comprises a group of permanent, non-progressive disorders affecting movement and posture, frequently associated with sensory, cognitive, and musculoskeletal impairments that significantly impact functional independence and quality of life [1,2,3].

Among its clinical subtypes, spastic diplegia predominantly affects the lower limbs and is commonly associated with impaired gait, reduced postural control, and altered motor coordination. These motor deficits arise from a complex interaction between spasticity, muscle weakness, impaired selective motor control, and secondary musculoskeletal adaptations [4,5]. As a result, children with spastic diplegia often exhibit inefficient gait patterns, increased energy expenditure, and reduced participation in daily activities [4,5].

Recent evidence emphasizes that functional impairment in CP cannot be explained solely by spasticity, but rather by deficits in motor control, trunk stability, and neuromuscular coordination [6,7,8]. Contemporary neurorehabilitation approaches therefore focus on integrated, task-oriented interventions targeting multiple components of motor function, including balance, gait, and functional mobility [4,5,9].

Exercise-based rehabilitation remains a cornerstone of pediatric cerebral palsy management, with substantial evidence supporting its role in improving gross motor function, gait performance, and postural control [3,5,9,10,11,12,13]. However, considerable heterogeneity persists regarding intervention intensity, treatment duration, rehabilitation protocols, and outcome measures across published studies [4,5,10,11,12,13]. In addition to the GMFM, other established performance-based measures, including walking tests such as the 6-Minute Walk Test and the Timed Up and Go test, have been used to assess complementary dimensions of functional mobility and walking capacity in children with cerebral palsy [12,13]. Consequently, direct comparison between rehabilitation strategies remains challenging, and the long-term sustainability of functional improvements has not yet been fully clarified [4,5,11,14]. Studies in other pediatric populations have also highlighted the value of exercise-based interventions and multidimensional functional assessment [15,16,17].

Despite these advances, important gaps remain in the evidence regarding the long-term evolution of functional outcomes following structured rehabilitation in children with cerebral palsy. Most available studies and systematic reviews primarily focus on short-term interventions or individual therapeutic approaches, whereas relatively few have prospectively evaluated the simultaneous evolution of gross motor function, muscle tone, and joint mobility during prolonged rehabilitation programs [10,11,12,14]. Furthermore, recent systematic reviews have highlighted substantial heterogeneity in rehabilitation protocols, intervention duration, and outcome measures, limiting direct comparisons across studies and emphasizing the need for standardized longitudinal assessments in pediatric neurorehabilitation [4,5,11,14]. Spasticity, joint mobility, and gross motor function were selected as complementary outcome domains because they reflect interconnected neuromuscular, biomechanical, and functional aspects of spastic cerebral palsy. Their concurrent assessment is clinically relevant because changes in muscle tone and joint mobility may influence functional performance, while gross motor function reflects the integration of these and other motor determinants into functional activity. In this context, the present study adds to the existing literature by concurrently tracking these complementary clinical and functional domains at three standardized time points over 12 months and by examining the longitudinal relationships between muscle spasticity and joint mobility. From a clinical perspective, this multidimensional approach may provide a more comprehensive characterization of functional evolution during sustained rehabilitation and may support the longitudinal monitoring and individualization of rehabilitation strategies in children with spastic diplegic cerebral palsy. Therefore, the aim of the present study was to prospectively evaluate the longitudinal evolution of gross motor function, muscle tone, joint mobility, and the relationships between muscle spasticity and joint mobility during a structured 12-month rehabilitation program in children with spastic diplegic cerebral palsy. By integrating repeated clinical and functional assessments throughout the rehabilitation period, the study sought to provide a comprehensive longitudinal characterization of functional recovery. Accordingly, the study addressed the following research questions: (1) How do motor function, muscle tone, and joint mobility change over the course of a structured 12-month rehabilitation program in children with spastic diplegic cerebral palsy? (2) Are changes in these clinical and functional outcomes sustained across the intermediate (6-month) and final (12-month) assessments? (3) What relationships exist between muscle spasticity and joint mobility, and how do these relationships evolve during the 12-month rehabilitation period?

It was hypothesized that (1) significant longitudinal improvements in motor function, muscle tone, and joint mobility would be observed over the 12-month study period and (2) these clinical and functional outcomes would show continued improvement between the intermediate (6-month) and final (12-month) assessments.

## 2. Materials and Methods

### 2.1. Study Design and Participants

This prospective longitudinal observational study included consecutive children with spastic diplegic cerebral palsy who were initially evaluated at the Department of Pediatric Surgery, County Emergency Clinical Hospital of Craiova, Romania, and subsequently enrolled in a structured 12-month rehabilitation program delivered through specialized Physical and Rehabilitation Medicine services. Between March 2018 and March 2022, a total of 116 children were screened for eligibility.

Children were eligible if they (1) were aged 5–10 years; (2) had a diagnosis of spastic diplegic cerebral palsy; (3) participated in the structured 12-month rehabilitation program; and (4) completed standardized clinical and functional assessments at baseline (T0), after 6 months (T1), and after 12 months (T2).

Children were excluded if they (1) had another subtype of cerebral palsy; (2) had incomplete clinical or functional data; (3) had severe cognitive or behavioral impairment preventing standardized assessment; (4) were scheduled for orthopedic surgery at the time of enrolment; (5) had undergone botulinum toxin injections within the 6 months preceding enrolment, were receiving baclofen treatment at the time of enrolment, had a planned botulinum toxin or baclofen treatment during the 12-month study period; or (6) failed to complete follow-up evaluations.

**Figure 1:**
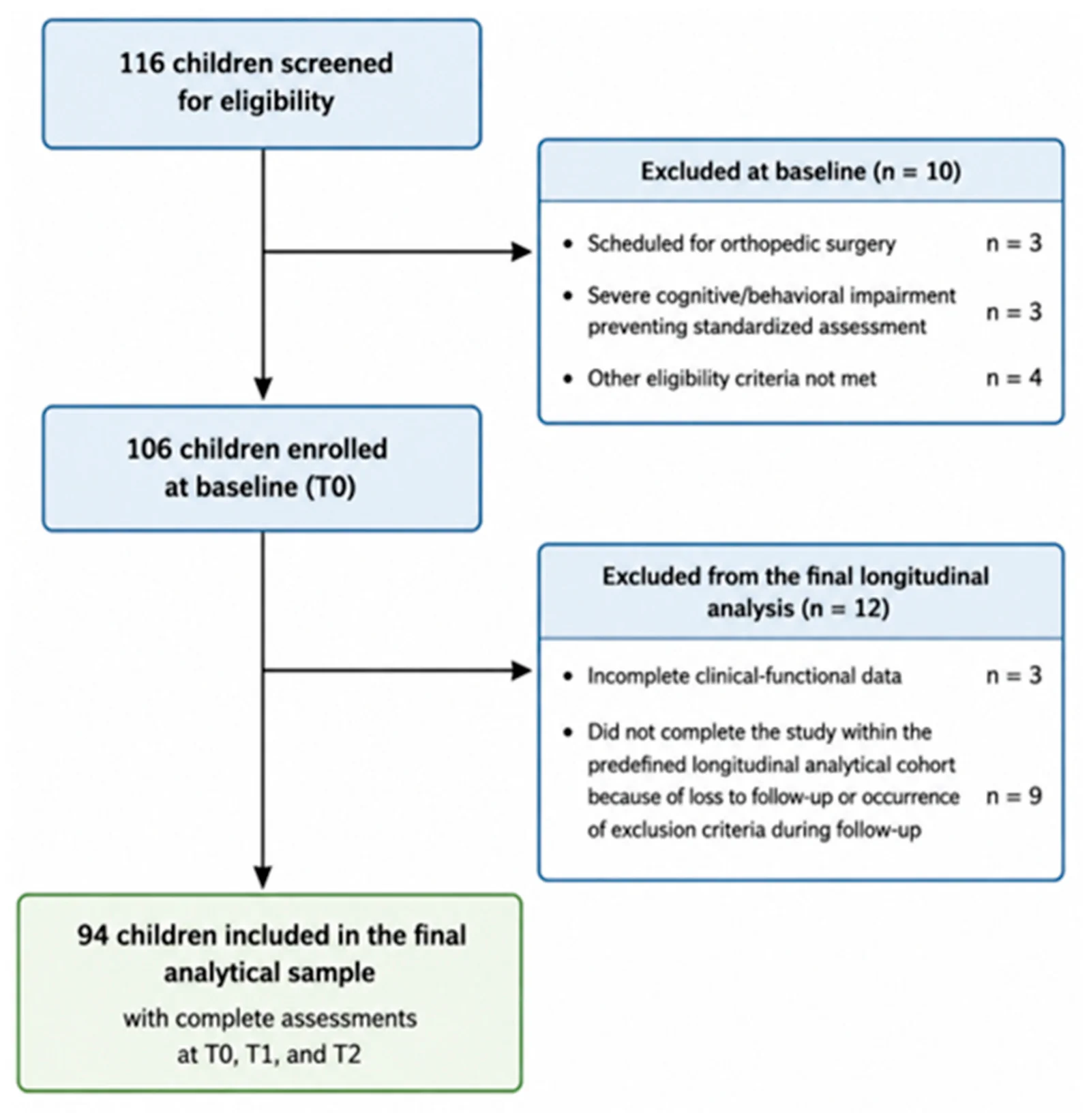
Flow diagram of participant screening, enrolment, follow-up, and inclusion in the final longitudinal analysis.

Of the 116 children screened for eligibility, 10 were excluded at baseline and 106 were enrolled in the longitudinal study. During follow-up, 12 participants were excluded from the final longitudinal analysis because of incomplete clinical-functional data (*n* = 3) or failure to complete the study within the predefined analytical cohort due to loss to follow-up or the occurrence of exclusion criteria during follow-up (*n* = 9). Consequently, the final analytical sample comprised 94 children with complete assessments at T0, T1, and T2 (Figure 1).

### 2.2. Study Population

Prior to enrolment, all children underwent a multidisciplinary clinical and functional evaluation to confirm the diagnosis of spastic diplegic cerebral palsy and verify eligibility for participation in the study. The selected age range was chosen to obtain a relatively homogeneous cohort within a defined developmental period and to reduce age-related variability in gross motor function. Gross motor development in children with cerebral palsy follows age-dependent trajectories, with the rate and extent of functional change varying throughout childhood [18,19]. The 5–10-year age interval therefore represents a clinically relevant developmental period for longitudinal assessment of motor function while limiting the heterogeneity associated with broader age ranges.

Participants for whom botulinum toxin injections or baclofen treatment were initiated during the 12-month follow-up were excluded from the longitudinal analytical cohort, as these interventions could independently modify muscle tone and potentially influence the clinical and functional outcomes under investigation. Hydrotherapy and electrotherapy were not exclusion criteria at baseline. However, participants who initiated either of these adjunct rehabilitation therapies during the 12-month follow-up were excluded from the final longitudinal analysis in order to reduce therapeutic heterogeneity and to limit the potential influence of additional rehabilitation interventions on the outcomes under investigation.

### 2.3. Ethics Approval

All participants and their parents or legal guardians were informed about the study objectives, procedures, and their role in the research. Particular attention was given to the protection of this vulnerable population, ensuring that all procedures were conducted with respect for participant safety, dignity, and confidentiality.

Written informed consent was obtained from the parents or legal guardians of all participants prior to inclusion in the study. Participants were informed of their right to withdraw from the study at any time without any consequences.

The study was conducted in accordance with the Declaration of Helsinki and Good Clinical Practice guidelines. Ethical approval was obtained from the Ethics Committee of University of Medicine and Pharmacy of Craiova, Romania, under approval number 196/11 December 2017. The study was not registered in a public clinical trial database.

### 2.4. Study Intervention

The rehabilitation program consisted of supervised kinesiotherapy sessions performed over five sessions per week for 12 months. Each session lasted approximately 40–45 min. The intervention was individualized according to each child’s functional status and focused on reducing lower-limb spasticity-related limitations and improving joint mobility, postural control, balance, gross motor function, and gait ability.

To reduce therapeutic heterogeneity, children who received additional hydrotherapy or electrotherapy during the study period were not included in the final analysis. The use of prescribed orthotic devices was permitted as part of standard clinical management for children with spastic diplegic cerebral palsy and was not considered an additional therapeutic modality. Individual orthotic use, including device type, frequency of use, and changes in orthotic management during the 12-month follow-up, was not systematically recorded; therefore, orthotic exposure could not be included as a separate variable in the analysis.

Rehabilitation Components:

The rehabilitation program was individualized according to each child’s functional status and targeted the main impairments associated with spastic diplegic cerebral palsy, including spasticity, reduced joint mobility, impaired postural control, balance deficits, and gait dysfunction. The intervention aimed to improve functional mobility, promote efficient movement patterns, and maximize independence in daily activities.

The intervention included a combination of therapeutic techniques aimed at improving motor function, postural control, and functional mobility [5,11,13,20,21]:

- Functional exercises: focused on improving gross motor function, coordination, and functional movement patterns;
- Postural and balance training: aimed at enhancing trunk stability, equilibrium, and spatial orientation;
- Gait training: designed to improve walking patterns, step control, and lower limb coordination;
- Stretching and joint mobility exercises: targeting muscle shortening and joint stiffness associated with spasticity;
- Muscle strengthening exercises: adapted to the child’s functional level to improve lower limb stability and support ambulation.

The general structure of a typical rehabilitation session, including the main phases and corresponding therapeutic objectives, is presented in Table 1.

### 2.5. Program Adaptation and Monitoring

Although the rehabilitation program was individualized according to each child’s clinical and functional profile, the overall treatment framework was standardized across participants. All children followed the same prescribed treatment frequency (five supervised sessions per week), session duration (40–45 min), and general session structure, incorporating mobility and stretching exercises, muscle strengthening, postural control, motor control and balance activities, gait training, and functional exercises. Individualization occurred primarily in the selection, difficulty, and progression of exercises within these predefined therapeutic domains rather than through substantial differences in the overall treatment framework.

Exercise selection, load, and progression were individualized according to each child’s motor impairment, muscle tone, joint mobility, muscle strength, motor control, balance, gait performance, functional abilities, and tolerance. Exercise load was not prescribed using fixed external resistance or a uniform number of repetitions. Progression was achieved by increasing task difficulty and active participation, reducing assistance when appropriate, and advancing toward more demanding functional activities.

The supervised kinesiotherapy intervention was likewise delivered throughout the 12-month study period by the same team of licensed physiotherapists, who followed the standardized rehabilitation framework described above.

To promote continuity of rehabilitation [11], parents received standardized instructions to encourage basic home-based exercises on non-treatment days. These recommendations included stretching of spastic muscle groups, maintenance of joint mobility through simple range-of-motion exercises, and encouragement of functional activities such as standing and walking. Adherence to these home-based recommendations was not formally monitored or quantified; therefore, individual exposure to home exercise could not be determined and was not included in the study analysis.

### 2.6. Standardized Assessment Protocol and Outcome Measures

Clinical and functional assessments were performed at baseline (T0), after 6 months (T1), and after 12 months (T2) by the same multidisciplinary rehabilitation team using standardized evaluation procedures.

T0 represented the baseline assessment at initiation of the rehabilitation program. The 6-month assessment (T1) was selected as an intermediate time point after a substantial period of exposure to the rehabilitation program, while the 12-month assessment (T2) corresponded to completion of the planned intervention period and allowed characterization of longer-term longitudinal changes.

- Validated clinical and functional outcome measures were selected to assess three complementary domains: gross motor function, muscle tone, and joint mobility. Gross Motor Function: Gross motor function was assessed using the Gross Motor Function Measure (GMFM-88), expressed as a percentage score (%), with higher scores indicating better motor performance. The GMFM is a validated instrument for assessing changes in gross motor function in children with cerebral palsy, with established reliability for repeated clinical assessment [22].
- Muscle Tone: Muscle tone was assessed using the Modified Ashworth Scale (MAS), expressed as ordinal scores ranging from 0 to 4, with higher scores indicating greater resistance to passive movement. Previous studies in children with spastic cerebral palsy have reported variable but generally moderate-to-good inter-rater reliability for the MAS, with reliability differing according to the muscle group assessed [23];
- Joint Mobility: Passive joint range of motion was measured bilaterally using standardized goniometric techniques and expressed in degrees (°). Hip abduction, knee extension deficit, and ankle dorsiflexion were evaluated to quantify movement limitations associated with spasticity. Previous studies in children with cerebral palsy have demonstrated clinically acceptable intra- and inter-rater reliability for standardized goniometric measurements, although measurement reliability may vary according to the joint, measurement protocol, and examiner [24].

### 2.7. Statistical Analysis

All data were recorded and processed using Microsoft Excel (Microsoft 365, Microsoft Corp., Redmond, WA, USA) and IBM SPSS Statistics version 26.0 (IBM Corp., Armonk, NY, USA).

The longitudinal statistical analysis was restricted to participants with complete clinical and functional data available at all three assessment time points (T0, T1, and T2), corresponding to a complete-case analysis. An intention-to-treat analysis was not applicable because this was a prospective observational study rather than a randomized controlled trial.

Continuous variables were expressed as mean ± standard deviation (SD), while categorical variables were presented as absolute frequencies and percentages. For variables with non-normal distribution, median and interquartile range (IQR) were also reported. Given the ordinal nature of the Modified Ashworth Scale, median values were considered the primary descriptive measures for interpretation; mean ± SD values were additionally reported to facilitate description of longitudinal trends and comparison across assessment points. For continuous outcome variables, 95% confidence intervals (CIs) for the mean were calculated at each assessment time point.

The normality of data distribution was assessed using the Shapiro–Wilk test. As most variables did not follow a normal distribution, non-parametric statistical methods were applied.

To evaluate changes over time within the same cohort across the three evaluation time points (T0, T1, and T2), the Friedman test was used for repeated measures. When the overall Friedman test indicated a statistically significant difference, post hoc pairwise comparisons were performed between the three assessment time points (T0 vs. T1, T0 vs. T2, and T1 vs. T2). A Bonferroni correction was applied to account for the three pairwise comparisons.

Associations between clinical and functional parameters at each assessment time point were assessed using Spearman’s rank correlation coefficient (ρ), due to the ordinal nature of some variables (e.g., Modified Ashworth Scale) and the non-normal distribution of the data. The strength of correlations was interpreted as follows: weak (ρ < 0.3), moderate (0.3–0.7), and strong (ρ > 0.7). A *p*-value of less than 0.05 was considered statistically significant for the overall analyses. For the three post hoc pairwise comparisons, a Bonferroni-adjusted significance threshold of *p* < 0.0167 was applied.

## 3. Results

### 3.1. Demographic and Clinical Characteristics

A total of 94 children diagnosed with spastic diplegic cerebral palsy were included in the final analysis. The study population consisted of 38 boys (40.43%) and 56 girls (59.57%). The age of the participants ranged between 5 and 10 years. The mean age of the participants was 6.47 ± 1.38 years (range, 5–10 years), with the majority of children being 6 years old (51.06%), followed by 5-year-old children (22.34%). The remaining age groups were less represented, with proportions below 10% for each category.

Regarding the living environment, most participants originated from urban areas (72.34%), while 27.66% were from rural settings. In terms of family status, the majority of children (87.23%) lived within a family environment, whereas 13.10% were institutionalized and 1.19% were in foster care. Muscle tone evaluation using the Modified Ashworth Scale indicated that most children presented moderate to severe spasticity, particularly at the level of the hip adductors, knee flexors, and plantar flexors. Joint mobility evaluation demonstrated limitations in hip abduction, knee extension, and ankle dorsiflexion, consistent with spastic motor impairment. Gross motor function assessment using the GMFM-88 scale revealed higher scores in basic motor domains such as lying and rolling, and lower scores in advanced functional activities, including standing, walking, running, and jumping. Detailed baseline demographic and clinical characteristics are presented in Table 2 and Table 3.

### 3.2. Muscle Tone

Muscle tone was evaluated using the Modified Ashworth Scale.

Modified Ashworth Scale scores showed a progressive decrease across all evaluated muscle groups, including the hip adductors, hamstrings, and gastrocnemius muscles, over the 12-month study period (Table 4).

**Figure 2:**
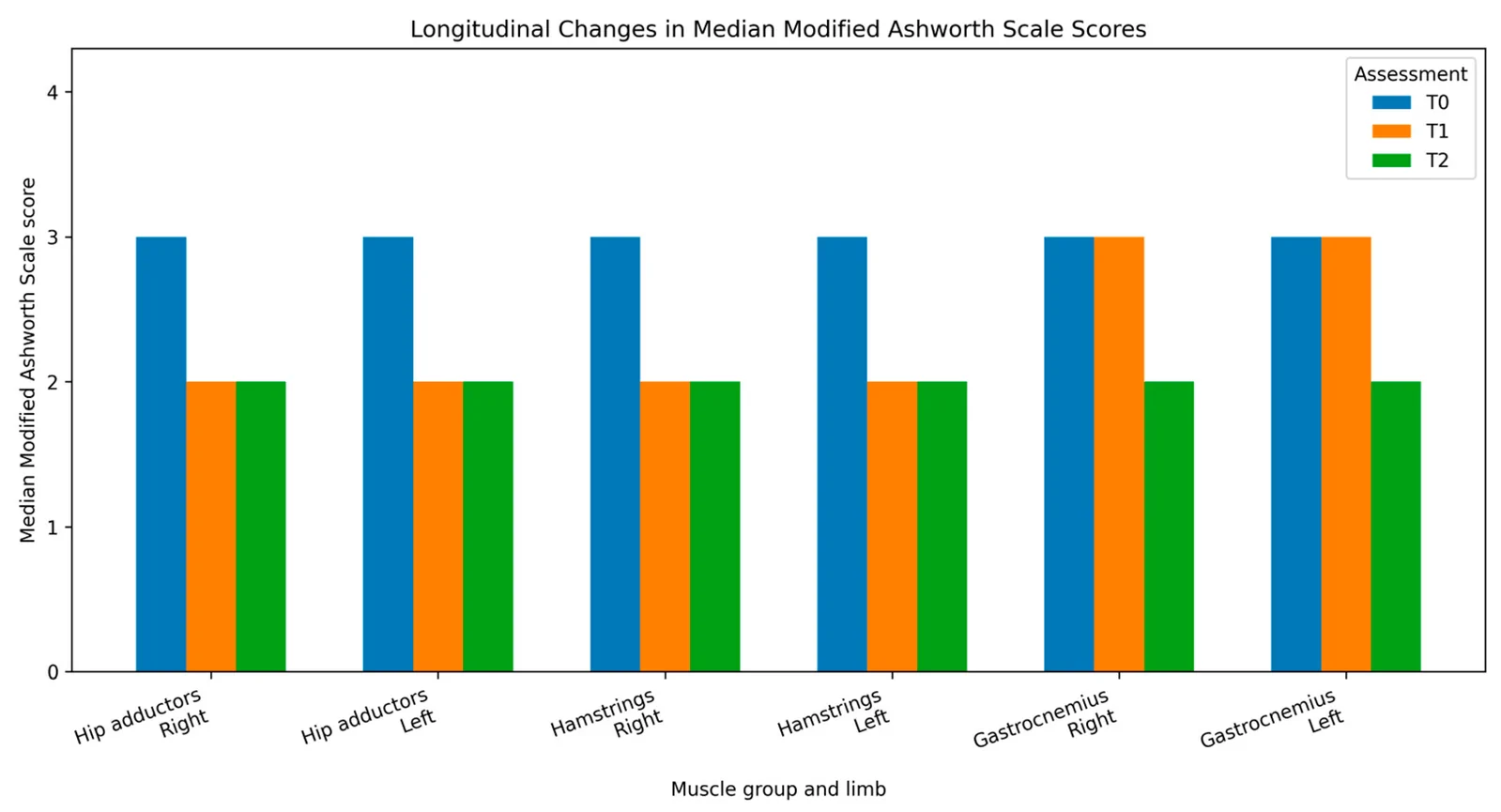
Longitudinal changes in median Modified Ashworth Scale scores across the three assessment time points.

The longitudinal evolution of median MAS scores across the evaluated muscle groups and both lower limbs is additionally illustrated in Figure 2.

Modified Ashworth Scale scores showed a progressive shift toward lower categories across the three assessment points. Median scores decreased from 3 at baseline to 2 at the final assessment in the evaluated muscle groups, indicating lower levels of spasticity at the group level over time. Mean ± SD values, reported as complementary descriptive measures, showed a similar longitudinal pattern across all muscle groups and both lower limbs. For example, gastrocnemius mean scores decreased from 3.33 ± 0.60 to 2.18 ± 0.58 for the right limb and from 3.30 ± 0.59 to 2.06 ± 0.50 for the left limb. The Friedman test showed significant differences across the three assessment time points (*p* < 0.0001). Post hoc pairwise comparisons were subsequently performed between T0 and T1, T0 and T2, and T1 and T2 using the Bonferroni-adjusted significance threshold of *p* < 0.0167.

### 3.3. Joint Mobility

Joint mobility parameters showed progressive improvement over the 12-month rehabilitation period. Mean values, together with their corresponding 95% confidence intervals, and changes over time are summarized in Table 5.

Values are expressed in degrees (°). SD = standard deviation; CI = confidence interval. The 95% CIs refer to the mean values at each assessment point. Negative values indicate movement limitation (dorsiflexion deficit or flexion contracture). T0 = baseline; T1 = 6 months; T2 = 12 months. Hip abduction increased in both lower limbs, with mean values rising from 29.85 ± 7.12° to 40.15 ± 9.06° for the left limb and from 28.03 ± 6.49° to 37.73 ± 9.11° for the right limb. Knee extension deficit decreased substantially over time, with mean values improving from 10.61 ± 5.83° to 1.36 ± 2.58° for the left limb and from 10.30 ± 5.99° to 1.36 ± 2.26° for the right limb, indicating a reduction in flexion contracture. Ankle dorsiflexion also improved, with mean values progressing from negative baseline values (−12.27 ± 7.40° left, −12.12 ± 7.50° right) to near-normal or positive values at the final evaluation (0.45 ± 5.50° left and −0.15 ± 5.79° right). Median values confirmed these trends, showing increased hip mobility, reduction in knee flexion deficit to near zero, and normalization of ankle dorsiflexion at the end of the rehabilitation program. From T0 to T2, the absolute mean changes were 9.70° and 10.30° for right and left hip abduction, respectively; −8.94° and −9.25° for right and left knee extension deficit, respectively; and 11.97° and 12.72° for right and left ankle dorsiflexion, respectively. These absolute changes provide an additional descriptive estimate of the magnitude of the observed longitudinal changes in joint mobility. The Friedman test showed significant differences over time (*p* < 0.0001). Following the significant overall Friedman test, post hoc pairwise comparisons of joint mobility parameters were performed between T0 and T1, T0 and T2, and T1 and T2, applying the Bonferroni-adjusted significance threshold of *p* < 0.0167.

### 3.4. Gross Motor Function (GMFM-88)

Mean GMFM-88 values and their corresponding 95% confidence intervals at each assessment point are presented in Table 6.

GMFM-88 = Gross Motor Function Measure; SD = standard deviation; CI = confidence interval; T0 = baseline; T1 = 6 months; T2 = 12 months. The 95% CIs refer to the mean GMFM-88 values at each assessment point. Mean values increased from 54.92 ± 1.65 at baseline to 57.99 ± 1.66 at 6 months and further to 61.52 ± 1.77 at 12 months. Median values showed a similar upward trend, increasing from 54.8 at T0 to 58.2 at T1 and 61.7 at T2. Minimum and maximum values also shifted toward higher ranges over time, consistent with the overall longitudinal trend observed at the group level. From T0 to T2, the mean GMFM-88 score increased by 6.60 points, corresponding to an approximately 12.0% increase relative to the baseline mean value. This absolute change provides an additional descriptive estimate of the magnitude of the observed longitudinal improvement in gross motor function. The Friedman test showed significant differences across the three assessment time points (*p* < 0.0001), indicating significant longitudinal differences in gross motor function over the study period. For GMFM-88 scores, pairwise comparisons were conducted between each assessment time point (T0 vs. T1, T0 vs. T2, and T1 vs. T2) following the significant Friedman test, with statistical significance evaluated using the Bonferroni-adjusted threshold of *p* < 0.0167.

### 3.5. Clinico-Mechanical Correlations

The analysis presented in this section focused on anatomically corresponding pairs of muscle spasticity and joint mobility parameters, as shown in Table 7.

A strong negative correlation was observed between hip abduction and spasticity of the hip adductors at all evaluation time points, with Spearman’s rank correlation coefficients (ρ) ranging from −0.675 to −0.831. This indicates that higher spasticity levels were associated with reduced hip abduction amplitude. For knee extension deficit and hamstring spasticity, moderate positive correlations were identified (ρ ranging from 0.489 to 0.682), suggesting that increased spasticity was associated with greater limitation in knee extension. Similarly, ankle dorsiflexion showed moderate-to-strong negative correlations with gastrocnemius spasticity, with coefficients ranging from −0.640 to −0.881, indicating that higher spasticity levels were associated with reduced dorsiflexion amplitude. Overall, these findings indicate consistent associations between muscle spasticity and joint mobility limitations at each assessment time point. As these correlations represent associations assessed separately at T0, T1, and T2, they should not be interpreted as evidence that reductions in spasticity caused the observed improvements in joint mobility.

## 4. Discussion

The present study evaluated the longitudinal functional outcomes of a structured 12-month rehabilitation program in children with spastic diplegic cerebral palsy, focusing on muscle tone, joint mobility, and gross motor function.

One of the most important findings of the present study was the progressive reduction in spasticity levels across all evaluated muscle groups, particularly in the gastrocnemius muscles, where baseline values were highest. This evolution should be interpreted as a gradual improvement over time rather than a strictly linear response, since the present analysis compared repeated evaluation moments but did not specifically test linear trend models. The observed reduction in spasticity is consistent with current evidence supporting active, goal-directed, and repetitive motor interventions in children with cerebral palsy, particularly when rehabilitation is individualized, intensive, and functionally oriented [3,4,25,26]. Contemporary evidence-based reviews emphasize the role of activity-based and task-specific approaches in cerebral palsy rehabilitation, with task-specific training showing potential benefits for motor performance, functional skills, and participation-related outcomes [5,27].

The reduction in spasticity observed in the present cohort was clinically relevant because it was accompanied by improvements in joint mobility and gross motor function. This association is supported by previous evidence demonstrating that spasticity is closely related to the development of muscle contracture and reduced range of motion, particularly at the level of the gastrosoleus–ankle complex [28]. In agreement with these findings, the inverse correlations identified in our study between gastrocnemius spasticity and ankle dorsiflexion, as well as between hip adductor spasticity and hip abduction, indicate associations between muscle tone and joint mobility at each assessment time point. These findings should not be interpreted as evidence that reductions in spasticity caused the observed improvements in joint mobility.

However, the relationship between spasticity and gross motor function remains complex. Spasticity alone explains only part of the variability in functional performance, as muscle strength, selective motor control, coordination, and secondary musculoskeletal changes also play a major role [29,30]. Therefore, the longitudinal data obtained should be interpreted within a broader framework of neuromotor adaptation associated with sustained rehabilitation.

An important contribution of the present study is the simultaneous longitudinal evaluation of spasticity, joint mobility, and gross motor function over a 12-month rehabilitation period in a relatively homogeneous cohort of children with spastic diplegic cerebral palsy.

Progressive improvements in joint mobility parameters were also observed over the 12-month rehabilitation period. Hip abduction increased significantly, while knee flexion deficit and ankle dorsiflexion limitation progressively decreased over time. These findings are clinically relevant because reduced joint mobility in spastic diplegia is strongly associated with gait dysfunction, postural instability, and contracture formation. Previous systematic reviews and meta-analyses have demonstrated that interventions including stretching, strengthening, functional mobility training, treadmill-based rehabilitation, and task-oriented exercise may improve lower-limb function and gross motor performance in children with bilateral spastic cerebral palsy [3,21,31].

In particular, interventions targeting muscle–tendon unit extensibility and active motor control appear to contribute to both increased range of motion and improved functional performance, especially at the level of the ankle and knee joints [3,21]. The improvements in ankle dorsiflexion observed in the present study are clinically relevant, as reduced dorsiflexion is a key biomechanical factor contributing to equinus gait and functional limitations in children with spastic diplegia [31].

These findings are compatible with the rationale for combining active and mobility-oriented components within a multimodal rehabilitation framework; however, the present observational design does not allow the contribution of individual intervention components to be determined. Interestingly, the association between gastrocnemius spasticity and ankle dorsiflexion became less pronounced across the three assessment time points. While a very strong inverse association was observed at baseline, its strength decreased at subsequent evaluations. This pattern may suggest that the relationship between spasticity and ankle mobility changes over time; however, the present data do not establish the mechanisms underlying this change. Factors such as muscle–tendon properties, motor control, muscle strength, natural development, or other neuromuscular and biomechanical characteristics may contribute and should be investigated in future studies. Compared with previous studies, the current results provide additional value by documenting the evolution of joint mobility parameters in parallel with spasticity reduction and functional improvement over a 12-month period. While many studies report short-term changes, fewer have evaluated the longitudinal interaction between tone, mobility, and function within a single rehabilitation framework.

The improvements observed across multiple joint-mobility parameters may be clinically relevant in the context of secondary musculoskeletal complications, including contracture development and joint deformities, which represent important targets of long-term surveillance in children with cerebral palsy [32]. However, the present study did not assess whether rehabilitation altered the development or progression of these complications. Gross motor function, assessed using the GMFM-88, also showed progressive improvement over the 12-month study period. The observed increase in GMFM-88 scores supports the presence of a longitudinal improvement in gross motor function at the group level. Similar improvements in motor function have been reported in systematic reviews evaluating active, task-oriented, and functionally oriented rehabilitation interventions in children with cerebral palsy [5,14,20].

Although statistically significant longitudinal differences were observed across the evaluated outcomes, statistical significance should be distinguished from clinical significance. Statistical significance indicates that the observed differences across assessment time points were unlikely to reflect random variation within the analyzed cohort, whereas clinical significance concerns whether the magnitude of change translates into meaningful functional improvement for individual patients. In the present study, the consistent longitudinal changes observed across muscle tone, joint mobility, and gross motor function suggest potential clinical relevance at the group level. However, because individual-level responder analyses and predefined thresholds for clinically meaningful change were not applied, the clinical significance of these findings should be interpreted cautiously and should not be inferred solely from statistical significance. The relatively low coefficient of variation observed for GMFM-88 scores may reflect the relatively homogeneous clinical profile of the study population. Participants were selected according to strict inclusion and exclusion criteria, including a single cerebral palsy subtype (spastic diplegia), a narrow age range (5–10 years), and completion of the same structured rehabilitation program. Consequently, variability in functional performance may have been lower than that reported in more heterogeneous cerebral palsy cohorts. However, changes in gross motor function do not necessarily occur in direct proportion to changes in spasticity or joint mobility, as functional performance in cerebral palsy is influenced by multiple interacting motor and biomechanical factors [5,29,30,31]. An important strength of the present study lies in the analysis of clinico-mechanical correlations. The identified relationships between spasticity and joint mobility provide quantitative support for the concept that functional limitation results from the interaction between abnormal muscle tone and secondary biomechanical restrictions [25,33]. The consistency of these correlations across all evaluation time points further strengthens their clinical relevance.

The study also has practical applicability in rehabilitation planning. The observed relationships between muscle tone and joint mobility may help clinicians prioritize therapeutic targets according to the predominant biomechanical limitation. The multidimensional evaluation model may also facilitate longitudinal monitoring of rehabilitation outcomes in clinical practice.

The internal validity of the study was strengthened by the prospective longitudinal design, standardized evaluation protocol, homogeneous clinical subtype selection, and repeated assessments.

However, several limitations should be acknowledged. Most importantly, the absence of a control group precludes causal attribution of the observed longitudinal changes to the rehabilitation program. Given the age of the participants, maturational changes and natural developmental progression may have contributed to the observed changes and cannot be separated from changes occurring during the rehabilitation period. In addition, the age distribution within the cohort was uneven, with most participants being 5–6 years old. Consequently, the longitudinal findings predominantly reflect younger children within the predefined 5–10-year age range and may be less representative of children toward the upper end of this age interval. The multimodal and individualized nature of the intervention represents an additional limitation, as the relative contribution of individual rehabilitation components cannot be determined. Moreover, individual orthotic use and potential changes in orthotic management during follow-up were not systematically recorded, precluding assessment of their potential influence on the observed outcomes. Home-based exercises were recommended to support continuity of care between supervised sessions; however, adherence was not systematically monitored or quantified. Consequently, variation in home-exercise exposure represents a potential unmeasured confounding factor. In addition, participants with incomplete follow-up data were excluded from the final analysis. Although complete longitudinal data were required for repeated-measures analysis, this exclusion may have introduced selection bias if participants who did not complete follow-up differed systematically from those retained in the study. Unmeasured co-interventions or changes in clinical management during the 12-month observation period also cannot be entirely excluded. The assessment protocol relied primarily on established clinical measures, including the GMFM-88, Modified Ashworth Scale, and standardized goniometry. Although these measures are widely used in the clinical evaluation of children with cerebral palsy, they do not provide the detailed quantitative biomechanical information obtainable from instrumented gait or movement analysis. This should be considered when interpreting the observed changes in muscle tone, joint mobility, and gross motor function. Finally, the relatively homogeneous cohort and restricted age range limit the generalizability of the findings to other age groups and cerebral palsy subtypes. Despite these limitations, the study provides valuable longitudinal data regarding the evolution of muscle tone, joint mobility, and gross motor function during prolonged rehabilitation. Compared with studies focused on short-term interventions, these findings provide additional information on the longitudinal evolution of these outcomes over a 12-month rehabilitation period.

Future studies should include controlled designs, larger multicenter cohorts, and objective biomechanical assessment tools to better clarify the long-term effects of rehabilitation strategies in children with cerebral palsy.

## 5. Conclusions

This study provides a longitudinal description of changes in muscle tone, joint mobility, and gross motor function over a 12-month period in a relatively homogeneous cohort of children with spastic diplegic cerebral palsy participating in a structured rehabilitation program. Progressive reductions in spasticity, improvements in joint mobility, and increases in GMFM-88 scores were observed across the three assessment time points, indicating favorable longitudinal changes at the group level.

The observed associations between muscle spasticity and joint mobility further illustrate the relationship between neuromuscular and biomechanical features in this population, without implying a causal relationship between changes in these parameters. Taken together, the findings provide supportive observational evidence for the clinical relevance of sustained, individualized rehabilitation and multidimensional longitudinal assessment in children with spastic diplegic cerebral palsy. However, given the absence of a control group, the observed changes cannot be attributed exclusively to the rehabilitation program, and the potential contributions of maturation, natural developmental progression, and other unmeasured factors cannot be excluded.

## Figures and Tables

**Table 1:** Structure of a typical kinesiotherapy session.

Phase	Objective	Type of Exercises
Warm-up (5–10 min)	Prepare the body for activity	Light mobilization, gentle active range-of-motion (ROM) exercises
Mobility training	Improve joint mobility and flexibility	Active and active-assisted ROM exercises, stretching, joint mobilization exercises
Strength training	Increase muscle strength	Isometric and isotonic exercises with resistance
Postural control	Improve alignment and trunk stability	Functional positioning, posture correction, trunk stabilization exercises
Motor control and balance	Enhance coordination and equilibrium	Balance exercises, repetitive task-oriented activities
Gait training	Improve walking ability	Gait re-education, assisted walking, step training, coordination drills
Cool-down (5 min)	Relaxation and recovery	Gentle stretching, breathing exercises

**Table 2:** Baseline demographic characteristics of the study population.

Characteristic	Value
Age (years), range	5–10
Age distribution, *n* (%) 5 years 6 years 7 years 8 years 9 years 10 years	21 (22.34%) 48 (51.06%) 3 (3.19%) 9 (9.57%) 8 (8.51%) 5 (5.32%)
Gender, *n* (%) Male Female	38 (40.43%) 56 (59.57%)
Residence, *n* (%) Urban Rural	68 (72.34%) 26 (27.66%)
Family status, *n* (%) Living with family Institutionalized Foster care	82 (87.23%) 11 (11.70%) 1 (1.06%)

*n* = number of patients; % = percent of patients.

**Table 3:** Baseline clinical characteristics of the study population.

Clinical Parameter	Clinical Characteristics	Value
Muscle tone (Modified Ashworth Scale) *n* (%)	Hip adductors	
	0–1+	29 (30.9%)
	2	34 (36.2%)
	3–4	31 (33.0%)
	Knee flexors	
	0–1+	26 (26%)
	2	34 (35%)
	3–4	34 (34%)
	Plantar flexors	
	0–1+	24 (24%)
	2	30 (33%)
	3–4	40 (43%)
Joint mobility (degrees) median (IQR)	Hip abduction	35 (31.3–40)
	Knee extension deficit	−10 (−15–0)
	Ankle dorsiflexion	0 (−10–0)
GMFM-88 median (IQR)	Section A (lying & rolling)	86 (80–90)
	Section B (sitting)	58 (38–63)
	Section C (crawling & kneeling)	63 (41–70)
	Section D (standing)	38 (11–43)
	Section E (walking, running, jumping)	11 (0–14)

*n* = number of patients; % = percentage of patients; IQR = interquartile range; MAS = Modified Ashworth Scale; GMFM-88 = Gross Motor Function Measure. For descriptive presentation of baseline muscle tone, MAS scores were grouped into three categories (0–1+, 2, and 3–4) and are presented as *n* (%). This grouping was used only to summarize the baseline distribution and did not represent a modification of the original MAS scoring system. Longitudinal analyses of muscle tone were performed using the original ordinal MAS scores.

**Table 4:** Evolution of spasticity in hip adductors, hamstrings, and gastrocnemius muscles assessed using the Modified Ashworth Scale.

Muscle Group	Limb	Parameter	T0	T1	T2
Hip adductors	Right	Mean ± SD	2.79 ± 0.60	2.27 ± 0.57	1.61 ± 0.56
		Median	3	2	2
		Min–Max	2–4	1–3	1–3
	Left	Mean ± SD	2.73 ± 0.63	2.18 ± 0.58	1.64 ± 0.65
		Median	3	2	2
		Min–Max	2–4	1–3	1–3
Hamstrings	Right	Mean ± SD	2.91 ± 0.38	2.24 ± 0.50	1.61 ± 0.65
		Median	3	2	2
		Min–Max	2–4	1–3	1–3
	Left	Mean ± SD	2.91 ± 0.38	2.18 ± 0.46	1.55 ± 0.51
		Median	3	2	2
		Min–Max	2–4	1–3	1–2
Gastrognemius	Right	Mean ± SD	3.33 ± 0.60	2.64 ± 0.65	2.18 ± 0.58
		Median	3	3	2
		Min–Max	2–4	1–4	1–3
	Left	Mean ± SD	3.30 ± 0.59	2.61 ± 0.61	2.06 ± 0.50
		Median	3	3	2
		Min–Max	2–4	2–4	1–3

SD = Standard Deviation; Modified Ashworth Scale (0–4): higher scores indicate increased spasticity; T0 = baseline; T1 = 6 months; T2 = 12 months. Given the ordinal nature of the Modified Ashworth Scale, median values were considered the primary descriptive measure, while mean ± SD values are provided as complementary descriptive information.

**Table 5:** Evolution of joint mobility parameters (hip abduction, knee extension deficit, and ankle dorsiflexion) during the rehabilitation program.

Parameter	Limb	Metric	T0	T1	T2
Hip abduction (°)	Right	Mean ± SD 95% CI	28.03 ± 6.49 26.70–29.36	31.82 ± 6.83 30.42–33.22	37.73 ± 9.11 35.86–39.60
		Median	25	30	35
		Min–Max	15–40	20–45	25–55
	Left	Mean ± SD 95% CI	29.85 ± 7.12 28.39–31.31	34.55 ± 7.22 333.07–36.03	40.15 ± 9.06 38.29–42.01
		Median	30	35	40
		Min–Max	15–45	25–50	30–60
Knee extension deficit (°)	Right	Mean ± SD 95% CI	10.30 ± 5.99 9.07–11.53	4.55 ± 4.90 3.55–5.55	1.36 ± 2.26 0.90–1.82
		Median	10	5	0
		Min–Max	0–25	0–15	0–5
	Left	Mean ± SD 95% CI	10.61 ± 5.83 9.42–11.80	5.61 ± 5.12 4.56–6.66	1.36 ± 2.58 0.83–1.89
		Median	10	5	0
		Min–Max	0–20	0–20	0–10
Ankle dorsiflexion (°)	Right	Mean ± SD 95%CI	−12.12 ± 7.50 −13.66–10.58	−7.12 ± 7.29 −8.61–5.63	−0.15 ± 5.79 −1.34–1.04
		Median	−10	−10	0
		Min–Max	−25–5	−20–10	−10–10
	Left	Mean ± SD 95% CI	−12.27 ± 7.40 −13.79–10.75	−6.97 ± 6.84 −8.37–5.57	0.45 ± 5.50 −0.68–1.58
		Median	−10	−10	0
		Min–Max	−25–0	−20–5	−10–10

**Table 6:** Evolution of gross motor function assessed using the GMFM-88 score during the rehabilitation program.

Parameter	T0	T1	T2
Mean ± SD	54.92 ± 1.65	57.99 ± 1.66	61.52 ± 1.77
95% CI	54.58–55.26	57.65–58.33	61.16–61.88
Median	54.8	58.2	61.7
Min–Max	51.7–57.8	53.8–61.1	57.9–65.3
Coefficient of variation (%)	3.01	2.86	2.88

**Table 7:** Clinico-mechanical correlations between joint mobility parameters and muscle spasticity (Spearman’s rank correlation coefficient, ρ).

Parameter Pair	T0 (ρ)	T1 (ρ)	T2 (ρ)
Hip abduction—Ashworth (hip adductors, RL)	−0.713 **	−0.769 **	−0.831 **
Hip abduction—Ashworth (hip adductors, LL)	−0.675 **	−0.721 **	−0.730 **
Knee extension deficit—Ashworth (hamstrings, RL)	0.556 **	0.682 **	0.565 **
Knee extension deficit—Ashworth (hamstrings, LL)	0.653 **	0.609 **	0.489 *
Ankle dorsiflexion—Ashworth (gastrocnemius, RL)	−0.851 **	−0.758 **	−0.731 **
Ankle dorsiflexion—Ashworth (gastrocnemius, LL)	−0.881 **	−0.642 **	−0.640 **

RL = right limb; LL = left limb; ρ = Spearman’s rank correlation coefficient. Negative values indicate an inverse relationship (higher spasticity associated with reduced joint mobility), while positive values indicate a direct relationship (higher spasticity associated with greater movement limitation). T0 = baseline; T1 = 6 months; T2 = 12 months; * *p* < 0.05; ** *p* < 0.01.

## Data Availability

Data are contained within the article.The original contributions presented in this study are included in the article. Further inquiries can be directed to the corresponding author.

## References

[1] Rosenbaum P., Paneth N., Leviton A., Goldstein M., Bax M., Damiano D., Dan B., Jacobsson B.M. (2007). A report: The definition and classification of cerebral palsy April 2006. Dev. Med. Child Neurol. Suppl..

[2] Oskoui M., Coutinho F., Dykeman J., Jetté N., Pringsheim T. (2013). An update on the prevalence of cerebral palsy: A systematic review and meta-analysis. Dev. Med. Child Neurol..

[3] Novak I., McIntyre S., Morgan C., Campbell L., Dark L., Morton N., Stumbles E., Wilson S.-A., Goldsmith S. (2013). A systematic review of interventions for children with cerebral palsy: State of the evidence. Dev. Med. Child Neurol..

[4] Faccioli S., Pagliano E., Ferrari A., Maghini C., Siani M.F., Sgherri G., Cappetta G., Borelli G., Farella G.M., Foscan M. (2023). Evidence-based management and motor rehabilitation of cerebral palsy children and adolescents: A systematic review. Front. Neurol..

[5] Novak I., Morgan C., Fahey M., Finch-Edmondson M., Galea C., Hines A., Langdon K., McNamara M., Paton M.C.B., Popat H. (2020). State of the Evidence Traffic Lights 2019: Systematic Review of Interventions for Preventing and Treating Children with Cerebral Palsy. Curr. Neurol. Neurosci. Rep..

[6] Curtis D.J., Butler P., Saavedra S., Bencke J., Kallemose T., Sonne-Holm S., Woollacott M. (2015). The central role of trunk control in the gross motor function of children with cerebral palsy: A retrospective cross-sectional study. Dev. Med. Child Neurol..

[7] Shumway-Cook A., Woollacott M.H. (2017). Motor Control: Translating Research into Clinical Practice.

[8] Anttila H., Autti-Rämö I., Suoranta J., Mäkelä M., Malmivaara A. (2008). Effectiveness of physical therapy interventions for children with cerebral palsy: A systematic review. BMC Pediatr..

[9] Gonzalez N.A., Sanivarapu R.R., Osman U., Latha Kumar A., Sadagopan A., Mahmoud A., Begg M., Tarhuni M., N Fotso M., Khan S. (2023). Physical Therapy Interventions in Children With Cerebral Palsy: A Systematic Review. Cureus.

[10] Liang X., Tan Z., Yun G., Cao J., Wang J., Liu Q., Chen T. (2021). Effectiveness of exercise interventions for children with cerebral palsy: A systematic review and meta-analysis of randomized controlled trials. J. Rehabil. Med..

[11] Merino-Andrés J., García de Mateos-López A., Damiano D.L., Sánchez-Sierra A. (2022). Effect of muscle strength training in children and adolescents with spastic cerebral palsy: A systematic review and meta-analysis. Clin. Rehabil..

[12] Moreau N.G., Bodkin A.W., Bjornson K., Hobbs A., Soileau M., Lahasky K. (2016). Effectiveness of Rehabilitation Interventions to Improve Gait Speed in Children With Cerebral Palsy: Systematic Review and Meta-analysis. Phys. Ther..

[13] Booth A.T.C., Buizer A.I., Meyns P., Oude Lansink I.L.B., Steenbrink F., van der Krogt M.M. (2018). The efficacy of functional gait training in children and young adults with cerebral palsy: A systematic review and meta-analysis. Dev. Med. Child Neurol..

[14] Cortés-Pérez I., Castillo-Pintor M.L.Á., Barrionuevo-Berzosa R., Piñar-Lara M., Obrero-Gaitán E., García-López H. (2025). Dual-Task Training Interventions for Cerebral Palsy: A Systematic Review and Meta-Analysis of Effects on Postural Balance and Walking Speed. Medicina.

[15] Popescu C., Matei D., Trăistaru R. (2024). The Role of Kinesiotherapy in Enhancing Physical Performance and Self-Esteem: A Prospective Observational Study in Obese Adolescents. Balneo PRM Res. J..

[16] Popescu C., Matei D., Amzolini A.M., Trăistaru M.R. (2025). Comprehensive Gait Analysis and Kinetic Intervention for Overweight and Obese Children. Children.

[17] Popescu C., Matei D., Amzolini A.M., Trăistaru M.R. (2024). Inflammation and Physical Performance in Overweight and Obese Schoolchildren. Life.

[18] Rosenbaum P.L., Walter S.D., Hanna S.E., Palisano R.J., Russell D.J., Raina P., Wood E., Bartlett D.J., Galuppi B.E. (2002). Prognosis for Gross Motor Function in Cerebral Palsy: Creation of Motor Development Curves. JAMA.

[19] Hanna S.E., Rosenbaum P.L., Bartlett D.J., Palisano R.J., Walter S.D., Avery L., Russell D.J. (2009). Stability and Decline in Gross Motor Function among Children and Youth with Cerebral Palsy Aged 2 to 21 Years. Dev. Med. Child Neurol..

[20] Zai W., Xu N., Wu W., Wang Y., Wang R. (2022). Effect of task-oriented training on gross motor function, balance and activities of daily living in children with cerebral palsy: A systematic review and meta-analysis. Medicine.

[21] Ryan J.M., Cassidy E.E., Noorduyn S.G., O’Connell N.E. (2017). Exercise interventions for cerebral palsy. Cochrane Database Syst. Rev..

[22] Russell D.J., Rosenbaum P.L., Cadman D.T., Gowland C., Hardy S., Jarvis S. (1989). The gross motor function measure: A means to evaluate the effects of physical therapy. Dev. Med. Child Neurol..

[23] Mutlu A., Livanelioglu A., Gunel M.K. (2008). Reliability of Ashworth and Modified Ashworth scales in children with spastic cerebral palsy. BMC Musculoskelet. Disord..

[24] Allington N.J., Leroy N., Doneux C. (2002). Ankle joint range of motion measurements in spastic cerebral palsy children: Intraobserver and interobserver reliability and reproducibility of goniometry and visual estimation. J. Pediatr. Orthop. B.

[25] Damiano D.L. (2006). Activity, activity, activity: Rethinking our physical therapy approach to cerebral palsy. Phys. Ther..

[26] Spittle A.J., Morgan C., Panteliadis C.P. (2025). Early Intervention for Children with Cerebral Palsy. Cerebral Palsy.

[27] Toovey R., Bernie C., Harvey A.R., McGinley J.L., Spittle A.J. (2017). Task-specific gross motor skills training for ambulant school-aged children with cerebral palsy: A systematic review. BMJ Paediatr. Open.

[28] Hägglund G., Wagner P. (2011). Spasticity of the gastrosoleus muscle is related to the development of reduced passive dorsiflexion of the ankle in children with cerebral palsy: A registry analysis of 2,796 examinations in 355 children. Acta Orthop..

[29] Park E.Y. (2018). Path analysis of strength, spasticity, gross motor function, and health-related quality of life in children with spastic cerebral palsy. Health Qual. Life Outcomes.

[30] Ross S.A., Engsberg J.R. (2007). Relationships between spasticity, strength, gait, and the GMFM-66 in persons with spastic diplegia cerebral palsy. Arch. Phys. Med. Rehabil..

[31] Willoughby K.L., Dodd K.J., Shields N. (2009). A systematic review of the effectiveness of treadmill training for children with cerebral palsy. Disabil. Rehabil..

[32] Hägglund G., Alriksson-Schmidt A., Lauge-Pedersen H., Rodby-Bousquet E., Wagner P., Westbom L. (2014). Prevention of dislocation of the hip in children with cerebral palsy: 20-year results of a population-based prevention programme. Bone Jt. J..

[33] Gage J.R., Schwartz M.H., Koop S.E., Novacheck T.F. (2009). The Identification and Treatment of Gait Problems in Cerebral Palsy.

